# Attitudes on euthanasia among medical students and doctors in Sri Lanka: a cross sectional study

**DOI:** 10.1186/s12910-021-00731-2

**Published:** 2021-12-07

**Authors:** H. M. M. T. B. Herath, K. W. S. M. Wijayawardhana, U. I. Wickramarachchi, Chaturaka Rodrigo

**Affiliations:** 1 Institute of Neurology, National Hospital of Sri Lanka, Colombo, Sri Lanka; 2 Department of Paediatrics, Faculty of Medicine, University of Colombo, Colombo, Sri Lanka; 3 Department of Anatomy, Faculty of Medicine, University of Moratuwa, Moratuwa, Sri Lanka; 4 Department of Pathology, School of Medical Sciences, UNSW Sydney, Sydney, NSW Australia

**Keywords:** Euthanasia, Sri Lanka, Cross sectional study, Physician assisted suicide

## Abstract

**Background:**

Euthanasia is a topic of intense ethical debate and it is illegal in most countries at present, including Sri Lanka. The aim of this descriptive cross-sectional study of medical students and practicing doctors was to explore the acceptance of euthanasia and physician assisted suicide (PAS), and factors influencing this opinion.

**Methods:**

A customised online questionnaire which explored opinions on euthanasia was administered to first and final year medical undergraduates in University of Colombo and practicing doctors with more than 5 years of work experience at The National Hospital of Sri Lanka. Attitudes on euthanasia and PAS were also assessed with the attitudes towards euthanasia (ATE) Scale, which is a 10-item questionnaire.

**Results:**

A total of 425 individuals responded (males: 178, 42%, age: median – 27 years), which included 143 (33.6%) first-year medical undergraduates, 141 (33.2%) final-year medical undergraduates and 141 (33.2%) practicing doctors. More participants (200, 47.1%) favoured legalizing euthanasia than those directly opposing it (110, 25.9%), but a significant proportion (27%) remained undecided. The mean scores of ATE questionnaire from the whole sample were generally unfavourable towards euthanasia/PAS. Accepting euthanasia as an option for oneself (*p* =  < 0.001) was the strongest predictor of favouring euthanasia/PAS or supporting its legalization.

**Conclusion:**

In this cross-sectional survey, more respondents supported legalisation of euthanasia in Sri Lanka than those openly opposing it. Yet, a significant minority that responded as “undecided” for legalisation, were more likely to have unfavourable ATE.

**Supplementary Information:**

The online version contains supplementary material available at 10.1186/s12910-021-00731-2.

## Background

Euthanasia which is derived from Greek meaning ‘good death’ defines a deliberate act by a physician to administer drugs with the explicit intention of ending a patients’ life. This is different from physician assisted suicide (PAS) where the physician prescribes lethal drugs to patient on their request, as means to commit suicide, rather than administering it oneself [1]. On other occasions physicians can withhold or withdraw life sustaining treatments (WLST) with patients agreement [2]. The legal and ethical acceptance of each of these scenarios are different across countries.

Euthanasia had been a topic of intense ethical debate from the times of ancient Greece and Rome and in modern history, Samuel Williams a non-physician proposed the use of morphine to end a patient’s life in 1870 [3]. Since then the concept of euthanasia has been mostly debated within the legal frameworks of industrialized countries [4]. These debates centre around the ethics of performing such an act, presumed violation of the Hippocratic oath, religious beliefs, sanctity of life, and the stories of suffering of patients with an incurables illnesses [3]. At present voluntary euthanasia (with patient’s consent) is legal in only few countries including Canada, New Zealand, Belgium, the Netherlands, Luxemburg, Colombia, Spain and some States of Australia [5]. PAS is allowed in few more jurisdictions such as Switzerland, and some States of USA [6, 7]. However, the acts permitted under the laws in these countries or states are not similar with some being more restrictive than others. Results from various surveys over the last two decades in multiple countries to assess the perspective and the attitudes on euthanasia among physicians (and medical students), public, patients and the relatives of dying patients have demonstrated much heterogeneity, suggesting such attitudes may be context (e.g., personal beliefs and opinions) and culture specific [8–12]. Furthermore, these surveys are mostly conducted in high income countries in Europe, North America or Oceania with a Judeo-Christian religious background and the findings cannot be generalised to low- and middle-income countries in Asia where cultural and religious affiliations are different.

In Sri Lanka, a predominantly Buddhist country of 21 million people in the Indian Ocean, which has a well-functioning and widely accepted allopathic healthcare system, no similar surveys to enquire about attitudes on euthanasia had been done previously. The topic of euthanasia and its variations are taught in the undergraduate curricula of all medical schools of the country, but the act itself and PAS remains illegal. WLST is accepted and allowed within ethical and legal framework for incurable terminal illnesses (with or without consent) where futility of treatment is agreed upon by the consensus of treating physicians. Requests from patients for WLST under these circumstances would also be respected. Based on our personal experience, there have been more enquiries from patients or their relatives regarding euthanasia in recent years. Therefore, assessing the attitudes toward euthanasia in the local setting among doctors and medical students (future practitioners) will be helpful in understanding the scope for a change in management options for patients with terminal illnesses in future.

The objectives of this study were to (a) assess the acceptance of euthanasia (voluntary) among practicing doctors, and first and final year medical undergraduates in Sri Lanka, and (b) examine their attitudes towards a range scenarios encompassing euthanasia, PAS and WLST using a previously validated questionnaire. We hypothesized that the attitude towards euthanasia will be more favourable with more clinical exposure and work experience.

## Methods

This is a descriptive cross-sectional study on attitudes regarding voluntary euthanasia among medical students and practicing doctors in Sri Lanka in 2021. The respondents were from the University of Colombo, Sri Lanka and its affiliated teaching hospital—The National Hospital of Sri Lanka in Colombo, Sri Lanka. Both these institutions are the largest in the country out of all medical schools and public sector hospitals, respectively. The undergraduate curricula of all medical schools in the country address the topic of euthanasia, but not in the first year of teaching. Therefore, we selected our undergraduate sample as first year and final year (the entire duration of undergraduate degree is 5.5 years) students to see if the attitudes were different after being formally taught about the topic, and after years of clinical exposure. Practicing doctors at the National Hospital of Sri Lanka were included if they had at least five years of clinical practice after internship. While the medical students were from a medical school of a single University, the practicing doctors in this survey had graduated from multiple universities in the country. Sri Lanka has only public sector Universities which offer undergraduate medical training, and they are all centrally regulated by the Ministry of Higher Education (except one military university under Ministry of Defence) in Sri Lanka. Therefore, the curricular contents are comparable. This study was approved by the Ethics Review Committee of the National Hospital of Sri Lanka.

The main data collection instrument was an anonymous customised online questionnaire administered in English language which collated data on participant demography (age, gender, religious and ethnic background), past experiences with euthanasia requests and individual opinion on legalisation of voluntary euthanasia. We also used the previously validated Attitudes Towards Euthanasia (ATE) Scale with permission from original authors [13]. This scale has 10 questions which explores attitudes towards a range of situations encompassing euthanasia (Q2, Q5, Q9) PAS (Q3, Q8) and WLST (Q1, Q4, Q6, Q7, Q10). The answer to each question is scored on a Likert scale, in response categories of (1) strongly disagree, (2) disagree, (3) undecided, (4) agree, and (5) strongly agree. We converted these to a numerical scale ranging from − 2 to 2 (with 0 for “undecided”) for purposes of analyses (a positive value indicates favourable attitudes to euthanasia). Questions 6 and 9 records a favourable attitude to euthanasia in reverse order compared to other questions, and for consistency the scoring was also reversed for these questions. For questions in WLST category the scenarios covered in Q1, Q7 are a grey area (WLST due to pain) and probably remains illegal in Sri Lanka while scenarios for Q4, Q6 and Q10 are legally allowed in Sri Lanka. Scenarios covered in all other questions (euthanasia and PAS) would be illegal under current laws. The ATE does not use the terms PAS or WLST in its explanations of scenarios covered by the questions and instead refers to outdated terms of “active” and “passive” euthanasia. We have instead used contemporary terms to describe the same scenarios as mentioned above. The questions only offer a scenario and do not use any of these technical terms, and therefore this change did not require a modification of the ATE. The answers for all questions were summed and compared across participants, in addition to responses to individual questions. The questionnaire was administered in English because it is the only language of teaching during undergraduate and postgraduate medical education in Sri Lanka. The language of professional communication among Sri Lankan doctors is also English. Since the ATE scale was not translated or modified, an internal validation was not done.

Each batch at the Colombo Medical faculty contains approximately 200 students and the number of doctors with five years of practice, working at the National Hospital of Sri Lanka was also comparable. While all eligible people in each group were invited to participate, the calculated sample size for a 95% confidence interval and a 5% margin of error was 132 per group (total of 396 participants). Data were analysed with Statistical Package of Social Science (SPSS, v25, IBM, USA). Descriptive statistics were summarised as measures of central tendency (mean or median) and dispersion (standard deviation or inter-quartile range) according to normality of distributions. The main outcomes were agreeing (or disagreeing) to legalise euthanasia (categorical variable). In the unadjusted analysis, continuous dependent variables were compared across categories with independent T test while two dependent variables were compared with linear regression, and two categorical variables were compared using the chi-square test. The adjusted analysis was completed with logistic and linear regression for categorical and continuous outcomes respectively. Cut-off for statistical significance was set at *p* < 0.05. In the analysis of the ATE, we summed up and averaged the scores of questions under following categories (Additional file 1: Table 1); euthanasia (Q2, Q5, Q9), PAS (Q3, Q8), WLST (Q1, Q4, Q6, Q7, Q10), legal scenarios (Q4, Q6, Q10), illegal scenarios (Q1, Q2, Q3, Q5, Q7, Q8, Q9). These averaged values were compared across the respondent groups defined in Table 1.

**Table 1:** Descriptive statistics of participant demography and their responses on euthanasia related questions

Category	Frequency and percentage (%)
*Experience and education*	
First-year medical undergraduate	143 (33.6)
Final-year medical undergraduate	141 (33.2)
Practicing doctors	141 (33.2)
*Gender*	
Male	178 (41.9)
female	247 (58.1)
*Religion*	
Buddhism	344 (80.9)
Catholicism/Christianity or Evangelical faith	22 (5.2)
Islam	15 (3.5)
Hindu	24 (5.6)
Atheist	16 (3.8)
Undisclosed	4 (0.9)
*Primary source of knowledge on euthanasia*	
Undergraduate/Post graduate curriculum	155 (36.4)
Seminars – professional and religious	128 (30.1)
Journal articles	108 (25.4)
Media/Internet	192 (45.1)
Peer education	125 (29.4)
*Opinion on legalising euthanasia*	
Yes	200 (47.1)
Undecided	115 (27.1)
No	110 (25.9)
*What illnesses should euthanasia be an option for*	
Terminal cancer	188 (44.2)
Degenerative neurological disease	177 (41.6)
End organ failure	129 (30.3)
Pain and suffering with any terminal illness	180 (42.3)
No response	82 (19.2)
Reasons for not favouring euthanasia*	
It is against my religious beliefs	103 (24.2)
It is against my conscience	115 (27.0)
Doctors should not interfere with the course of the nature	81 (19.0)
It is a violation of the Hippocratic oath	45 (10.5)
It is equivalent to murder	71 (16.7)
*Experience of past requests for euthanasia by a patient*	
First-year undergraduate	5 (3.4%)
Final-year undergraduate	40 (28.3%)
Practicing doctors	47 (33.3%)
*Self-acceptance of euthanasia*	
Yes	312 (73.4)
No	113 (26.6)
*Circumstances for self-acceptance of euthanasia*	
Loss of dignity	46 (14.7)
Loss of bodily function	123 (37.8)
Pain	184 (56.6)
Being dependent on others	185 (56.9)
Loss of meaning in life	134 (41.2)
Other	60 (18.4)

*Some individuals who were undecided on legalising euthanasia, still answered this question

## Results

A total of 425 people responded (males: 178, 42%, age: median – 27 years) which included 143 (33.6%) first-year medical undergraduates, 141 (33.2%) final-year medical undergraduates and 141 (33.2%) practicing doctors. Majority of respondents (344, 81%) identified themselves as Buddhists (Table 1). The response rate was 71–72% for students, and 53% for doctors. Overall, 200 participants (47.1%) favoured legalising euthanasia in Sri Lanka while 110 (25.9%) were opposed to such a move. Interestingly another 115 (27.1%) were undecided. In the unadjusted analysis (Table 2), being an atheist (*p* = 0.016), a practicing doctor (*p* = 0.004), having had a euthanasia request in the past (*p* =  < 0.001), being open to the option of euthanasia for oneself (*p* =  < 0.001) were associated with favouring legalisation of euthanasia. However, in the adjusted analysis (logistic regression), accepting euthanasia for oneself (*p* =  < 0.001) was the only independent predictor of favouring legalising of euthanasia.

**Table 2:** Associations with favouring legalisation of euthanasia

Total number responding in each category	Number favouring legalisation	*p* value
*Gender*		
Males (N = 132)	87	0.659
Females (N = 178)	113	
*Age (median – 27 years)*		
Above median (N = 140)	93	0.714
Below median (N = 163)	105	
*Being an atheist*		
Yes (N = 15)	14	0.016
No (N = 291)	183	
*Being a Buddhist*		
Yes (N = 238)	148	0.600
No (N = 53)	35	
Work and study experience**		
First year student (N = 89)	46	0.004
Final year student (N = 110)	82	
Practicing doctor (N = 111)	72	
*Having had a euthanasia request in the past*		
Yes (N = 79)	65	< 0.001
No (N = 231)	135	
*Acceptance of euthanasia for oneself*		
Yes (N = 219)	189	< 0.001*
No (N = 91)	11	

* = Significantly associated with favouring legalisation of euthanasia in the adjusted analysis (logistic regression), **Those who were undecided were excluded

Regarding the ATE questionnaire (Table 3), the summed average to each question and the whole tool were mostly in negative values (Additional file 1: Table 1) indicating that the majority of responses were not in favour of the scenarios presented in these questions. Scores were significantly more unfavourable towards illegal activities than legal activities in current context (mean score − 0.638 vs − 0.452, *p* = 0.001). Similarly, scenarios on euthanasia were significantly less favoured than those on PAS (mean score − 0.765 vs − 0.412, *p* < 0.0001). Scenarios on WLST scored in between and were also significantly different from mean scores for euthanasia or PAS scenarios (*p* < 0.05). Self-acceptance of euthanasia as an option for oneself were independently associated with a higher ATE score, but being a practicing doctor or a senior medical student did not influence it in the adjusted analysis, as hypothesized by us initially (Table 3). Findings were similar when the ATE questions were grouped according to scenarios they represent (Euthanasia, PAS or WLST) with self-acceptance euthanasia being the strongest independent predictor of favourable attitudes towards any one of these scenarios in the adjusted analysis (*p* < 0.05, Table 4). In addition, being an atheist was also independently associated with having favourable attitudes towards PAS and WLST (*p* < 0.05, Table 4), but not euthanasia. Interestingly, when ATE questions were re-grouped according to scenarios that would be legal in Sri Lanka or not, being an atheist was only associated with favouring the legal scenarios (*p* < 0.05, Table 4), while self-acceptance of euthanasia was independently associated with favouring both legal and other scenarios (*p* < 0.05, Table 4).

**Table 3:** Associations with the total score of the Attitudes Towards Euthanasia (ATE) scale

Category	Mean total ATE score (SD)	*p* value
*Gender*		
Male (N = 178)	− 6.15 (8.45)	0.455
Female (N = 247)	− 5.58 (6.76)	
*Age*		
> median (N = 176)	− 5.36 (7.94)	0.355
< median (N = 239)	− 6.05 (7.16)	
*Being an atheist*		
Yes (N = 16)	− 0.50 (8.31)	0.004
No (N = 405)	− 6.03 (7.49)	
*Being a Buddhist*		
Yes (N = 344)	− 5.79 (7.38)	0.111
No (N = 61)	− 7.43 (7.38)	
*Work and study experience*		
First year student (N = 143)	− 6.83 (7.41)	0.14
Final year student (N = 141)	− 5.28 (6.93)	
Practicing doctor (N = 141)	− 5.33 (8.03)	
*Having had a euthanasia request in the past*		
Yes (N = 92)	− 3.96 (7.00)	0.007
No (N = 333)	− 6.33 (7.57)	
*Being open to the option of euthanasia for oneself*		
Yes (N = 312)	− 3.55 (6.48)	< 0.001*
No (N = 113)	− 12.07 (6.55)	

*Had a significant association in the adjusted analysis

**Table 4:** Mean differences in attitudes towards euthanasia (ATE) scores when suggested questions were grouped according to different themes based on method of ending life and their legal status in Sri Lanka (the number of respondents in each category are the same as in Table 3)

Variable			Euthanasia*			PAS*				WLST*				Legal actions*				Illegal actions*		
			Mean (SD)	*p* value		Mean (SD)		*p* value		Mean (SD)		*p* value		Mean (SD)		*p* value		Mean (SD)		*p* value
Gender				NS				NS				NS				NS				NS
Male			–			–				–				–				–		
Female			–			–				–				–				–		
Age				NS				NS				NS				0.005				NS
Above median			–			–				–				− 0.32 (0.89)				–		
Below median			–			–				–				− 0.55 (0.82)				–		
Being an atheist				NS				0.007**				0.002**				0.001**				0.014
Yes			–			0.28 (1.24)				0.05 (0.91)				0.25 (0.94)				− 0.18 (0.81)		
No			–			− 0.44 (1.04)				− 0.56 (0.77)				− 0.48 (0.85)				− 0.66 (0.76)		
Being Buddhist				NS				NS				NS				NS				NS
Yes			–			–				–				–				–		
No			–			–				–				–				–		
Experience				NS				NS				0.042				0.002				NS
Student – first year			–			–				− 0.67 (0.79)				− 0.64 (0.86)				–		
Student – last year			–			–				− 0.49 (0.74)				− 0.43 (0.79)				–		
Doctor			–			–				− 0.46 (0.8)				− 0.29 (0.91)				–		
Past requests for euthanasia				0.01				NS				0.005				0.003				0.02
Yes			− 0.57 (0.84)			–				− 0.34 (0.7)				− 0.21 (0.83)				− 0.47 (0.72)		
No			− 0.82 (0.79)			–				− 0.6 (0.79)				− 0.52 (0.86)				− 0.68 (0.77)		
Self-acceptance of euthanasia				< 0.001**				< 0.001**				< 0.001**				< 0.001**				< 0.001**
Yes			− 0.58 (0.76)			− 0.1 (0.94)				− 0.32 (0.68)				− 0.23 (0.78)				− 0.41 (0.67)		
No			− 1.27 (0.73)			− 1.27 (0.88)				− 1.15 (0.7)				− 1.05 (0.79)				− 1.27 (0.66)		

*Grouping of questions are as follows; Euthanasia—Q2, Q5, Q9; PAS (Physician assisted suicide)—Q3, Q8; WLST (withholding or withdrawing life-sustaining treatment)—Q1, Q4, Q6, Q7, Q10; Legal actions in Sri Lanka—Q4, Q6, Q10; Illegal actions in Sri Lanka—all others except Q4, Q6, Q10, **Significalt asscocation in the adjusted analysis

## Discussion

In this survey of first- and final-year medical undergraduates and practicing doctors in Sri Lanka, just under half of the participants favoured legalization of euthanasia while a little more than a quarter were undecided. Accepting euthanasia as an option for oneself was the strongest predictor for favouring legalisation. Interestingly when the attitudes towards euthanasia, PAS or WLST were further explored with a validated scale (ATE scale), the overall trend of responses were largely unfavourable to all these scenarios indicating that those who responded as “undecided” on legalisation were more likely to have unfavourable attitudes.

As shown in the responses, even the first-year medical students were knowledgeable of euthanasia though they had not been exposed to formal teaching on the topic in the undergraduate curriculum. Sources for this “informal” knowledge was mostly media and internet. As expected, significantly more final-year undergraduates and practicing doctors favoured legalisation of euthanasia than first-year medical students. This suggests that formal teaching on the topic or clinical and personal experiences in the older groups of respondents may have had a significant effect in favour of accepting euthanasia. However, such a relationship was not observed when the ATE scores were analysed between the same groups. The significant minority that responded as “undecided” to the question on legalisation (hence excluded from that analysis), responded to the ATE questions and this may be the reason for the conflicting result. This subgroup was not entirely neutral and instead had more unfavourable attitudes towards euthanasia, as reflected in the summed aggregate for each ATE question. This was also evident from the higher-than-expected response rate (more than the number of people who said “no” to legalising) to the question which asked for reasons for not supporting legalisation of euthanasia. This observation also demonstrates the value of using a standardised scale to assess attitudes towards euthanasia since at first glance those supporting legislation seemed outnumbered those not supporting it, but when digging deep the majority of respondents actually had unfavourable attitudes towards the subject. These results are in contrast to similar surveys conducted in Australia [14], South India [15] and Israel [16], where a majority of respondents agreed for legislation. However, personal opinions may change with time and a study of medical students in Austria over 9 years showed that acceptance of active euthanasia increased from 16.3 in 2001 to 49.5% in 2009 [17]. Similarly, a study among physicians in Sweden in 2007 [18] showed that only 35% of respondents supported PAS (vs. 73% of public supporting the same at that time) [19], but this frequency increased to 47% when a follow-up survey was done among physicians in 2020 [20]. Interestingly in our study when ATE scores were grouped according to the scenario they represent, scores towards PAS were significantly more favourable than those for euthanasia while WLST was in between. Both euthanasia and PAS are illegal according to Sri Lankan law while the legally permissible WLST was perceived significantly less favourably than PAS. This is probably because the scenarios presented in WLST questions were a mix of legal and some “grey area” situations which are still probably illegal in Sri Lanka. For example, WLST for severe pain in a non-terminally ill patient might have led to a conflict of opinions when there are alternatives for pain relief. As expected, legal scenarios in ATE received significantly higher (favourable) scores than the illegal scenarios.

The strongest predictor for legalising euthanasia was being open to this option for oneself, indicating that respondents were more likely formulate decisions affecting others based on their own experiences and values. This observation was confirmed with the ATE score analysis as self-acceptance of euthanasia was the strongest independent predictor of having an overall high ATE score, favouring euthanasia, PAS or WLST, legal as well as illegal scenarios (according to Sri Lankan law) presented within the questionnaire.

Being an atheist (vs. religious) was significantly and independently associated with favouring PAS and WLST scenarios, but not Euthanasia scenarios in ATE. Religiosity, belief in afterlife and heaven, and religious denomination have been significantly associated with opposition to euthanasia previously [2, 15, 21]. Apart from religious affiliations, moral righteousness, slippery-slope argument, concerns on limitations on free-will of physicians, and doubts on the capacity of a dying patient to make an informed decision are other reasons cited by those opposing euthanasia in previous studies. On the other hand, pro-euthanasia sentiments are mainly based on relief of suffering and respecting patient autonomy [11, 22]. Only a few individuals identified themselves as atheists while a lot more accepted euthanasia as an option for oneself (Table 1). Hence the influence these two significant factors (atheism and self-acceptance of euthanasia) are likely to be independent of each other as influencers of attitudes towards euthanasia/PAS/WLST.

Systematic reviews have investigated factors determining the acceptance of euthanasia/PAS from the perspectives of patients, physicians (or medical students) and carers. A review of 17 studies (in 4 studies patients actually confronted end-of-life decisions) that recruited older patients found that (relatively) younger age, less religiosity, better education and higher socioeconomic status to be more consistently associated with acceptance of euthanasia and PAS, but there was high heterogeneity across the primary studies in design and results [8]. Regarding the carer’s perspective, a systematic review of studies from 4 countries (Canada, United States, Switzerland and the Netherlands) found that support from family members to be influenced by the legal status and social “acceptability” of PAS or euthanasia in each country. From the perspective of healthcare workers (nurses and doctors), a review of 27 studies (22 of these were from countries where euthanasia was illegal) showed that patient age, mental health of patient, medical speciality (for doctors) and past experience to be major factors leading to favourable attitudes towards euthanasia.

It is not uncommon for a physician to encounter requests for euthanasia during his or her career as shown in this study. A previous study in United kingdom which sampled general practitioners and consultants in the National Health Service found that approximately 60% of respondents had received requests for active or passive euthanasia from patients [2]. Studies from other countries put this percentage between 30 and 50% [14, 23]. Given that euthanasia or PAS is illegal, the uniform response for such requests in Sri Lanka would be a “no”. However, as people live longer and non-communicable diseases such as cancer become more common, requests for euthanasia will be more often encountered by future doctors in low- and middle-income countries including Sri Lanka. Therefore, next generations of physicians will need to reflect on one’s own attitudes regarding this issue, be prepared to have this discussion among professional colleagues and then with legislators and general public when arguments for or against legalisation come up. Even if majority sentiment of healthcare workers is unfavourable towards euthanasia or PAS, alternatives such as developing well-functioning palliative care services will need to be actively pursued as a compromise. Unfortunately, palliative care is not an independent subspeciality in Sri Lanka yet.

This study has several limitations. It only sampled from one medical school and one hospital in the country and hence the generalisability of results is limited. However, doctors working at the National Hospital of Sri Lanka would have graduated from different medical schools in the country and their participation may be representative of other medical schools in the country. This study by its design cannot observe changes in attitude in the same cohort over time and groupings based on different stages of training and career are only a surrogate for this purpose (changing attitudes with experience). If the same cohort of first-year undergraduates were followed up for 10 years, the findings may turn out to be different. In the groupings, we excluded doctors who had been in practice for less than 5 years because this ensured a 5-year gap in training for each of the groups which in our opinion was an adequate time window for attitudes to change. However, this time gap was selected arbitrarily. The ATE questionnaire as explained by original authors do not mention of PAS or WLST (instead it refers to “active” and “passive” euthanasia which some would consider to be outdated terms). We have considered the scenarios each question represents and grouped them as such for our analysis. However, we did not modify the original questionnaire when it was administered to participants.

## Conclusion

In conclusion, this cross-sectional survey of medical students and practicing doctors in Sri Lanka showed that more respondents supported legalisation of euthanasia than those openly opposing it. Yet, a significant minority were “undecided” with largely unfavourable attitudes towards euthanasia. Accepting euthanasia as an option for oneself was the strongest predictor of supporting euthanasia, PAS or WLST rather than work/clinical exposure.

## Supplementary Information

**Additional file 1**. Summary of responses to the Attitudes Towards Euthanasia (ATE) scale.

## Data Availability

The data set for this publication is available upon request from the authors.

## Abbreviations

ATE: Attitudes towards euthanasia
PAS: Physician assisted suicide
WLST: Withdrawing or withholding life sustaining treatment
