# Supplementary material for: Attitudes on euthanasia among medical students and doctors in Sri Lanka: a cross sectional study
Source: BMC Med Ethics. 2021 Dec 7;22:162. doi: 10.1186/s12910-021-00731-2 (PMC8650524; doi:10.1186/s12910-021-00731-2)
Supplement: Supplementary file 1 — Additional file 1. Summary of responses to the Attitudes Towards Euthanasia (ATE) scale. [file 12910_2021_731_MOESM1_ESM.docx]

**Supplementary Tables**

Supplementary Table 1. Summary of responses to the Attitudes Towards Euthanasia (ATE) scale

| Question | Range | Scenario assessed* | Mean (standard deviation) |
| --- | --- | --- | --- |
| Q 1 | -2 to 2 | WLST - Illegal | - 0.37 (1.14) |
| Q 2 | -2 to 2 | Euthanasia | - 1.00 (1.03) |
| Q 3 | -2 to 2 | PAS | - 0.59 (1.11) |
| Q 4 | -2 to 2 | WLST - Legal | - 0.72 (1.17) |
| Q 5 | -2 to 2 | Euthanasia | - 0.92 (1.04) |
| Q 6 | -2 to 2 | WLST - Legal | - 0.63 (1.16) |
| Q 7 | -2 to 2 | WLST - Illegal | - 0.97 (0.94) |
| Q 8 | -2 to 2 | PAS | - 0.24 (1.19) |
| Q 9 | -2 to 2 | Euthanasia | - 0.38 (1.17) |
| Q 10 | -2 to 2 | WLST - Legal | 0.00 (1.22) |
| Sum | -20 to 20 |  | - 5.82 (7.55) |

*All scenarios regarding euthanasia and PAS are currently not legally allowed in Sri Lanka, Some scenarios presented for WLST are also illegal
